# Doppler Broadening of Neutron Cross-Sections Using Kaniadakis Entropy

**DOI:** 10.3390/e24101437

**Published:** 2022-10-09

**Authors:** Willian Vieira de Abreu, João Márcio Maciel, Aquilino Senra Martinez, Alessandro da Cruz Gonçalves, Lucas Schmidt

**Affiliations:** Instituto Alberto Luiz Coimbra de Pós-Graduação e Pesquisa em Engenharia (COPPE/UFRJ), Programa de Engenharia Nuclear (PEN), Universidade Federal do Rio de Janeiro, Rio de Janeiro 21941-914, RJ, Brazil

**Keywords:** Kaniadakis, *κ*-statistics, neutron cross-section, Doppler broadening function, Faddeeva function

## Abstract

In the last seven years, Kaniadakis statistics, or κ-statistics, have been applied in reactor physics to obtain generalized nuclear data, which can encompass, for instance, situations that lie outside thermal equilibrium. In this sense, numerical and analytical solutions were developed for the Doppler broadening function using the κ-statistics. However, the accuracy and robustness of the developed solutions contemplating the κ distribution can only be appropriately verified if applied inside an official nuclear data processing code to calculate neutron cross-sections. Hence, the present work inserts an analytical solution for the deformed Doppler broadening cross-section inside the nuclear data processing code FRENDY, developed by the Japan Atomic Energy Agency. To do that, we applied a new computational method called the Faddeeva package, developed by MIT, to calculate error functions present in the analytical function. With this deformed solution inserted in the code, we were able to calculate, for the first time, deformed radiative capture cross-section data for four different nuclides. The usage of the Faddeeva package brought more accurate results when compared to other standard packages, reducing the percentage errors in the tail zone in relation to the numerical solution. The deformed cross-section data agreed with the expected behavior compared to the Maxwell–Boltzmann.

## 1. Introduction

Over the last 20 years, the Kaniadakis entropy [1] and its power-law tailed statistical distributions have been applied in many different fields, such as finance [2], astrophysics [3,4,5,6], game theoretical equilibrium [7], gravitational physics [8,9], dusty plasma [10] and so many others. 

In nuclear reactor physics, 2015 marked the first idealization of applying the κ-deformed statistics, intending to describe situations in non-thermal equilibrium inside a nuclear reactor, with the first article on this being published in 2017 [11]. The Doppler broadening function is utilized to represent the thermal nuclear movement. This function is commonly considered with a medium in thermal equilibrium with a temperature of T and using the Maxwell–Boltzmann distribution to describe the random velocities of the nuclei. However, to comprehend situations that lie outside the thermal equilibrium, Guedes et al. [11] proposed a very new expression for a deformed Doppler broadening function considering the Kaniadakis statistics:

(1) $$\psi_{\kappa }\left(\xi ,x\right)=\frac{\xi }{2\sqrt{\pi }}B\left(\kappa \right)\int_{-\infty }^{+\infty }\frac{1}{1+y^{2}}i\exp_{\kappa }\left[\frac{-\xi^{2}{\left(x-y\right)}^{2}}{4}\right]dy,$$

where,

(2) $$x\equiv \frac{2}{\Gamma }\left(E-E_{0}\right);$$

(3) $$\xi \equiv \frac{\Gamma }{{\left(\frac{4E_{0}K_{B}T}{A}\right)}^{\frac{1}{2}}};$$

κ is a deviation parameter that measures the deviation concerning the Maxwell–Boltzmann distribution [1,12], $k_{B}$ is the Boltzmann constant, $E_{0}$ is the resonant energy, *E* is the energy of the incident neutron, *A* is the mass number, and Γ is the total width of the resonance as measured in the laboratory coordinates. Furthermore,

(4) $$y\equiv \frac{2}{\Gamma }\left(E_{CM}-E_{0}\right);$$

(5) $$B\left(\kappa \right)={\left(2\left|\kappa \right|\right)}^{\frac{3}{2}}\left(1+\frac{1}{2}3\left|\kappa \right|\right)\frac{\Gamma \left(\frac{1}{2\left|\kappa \right|}+\frac{3}{4}\right)}{\Gamma \left(\frac{1}{2\left|\kappa \right|}-\frac{3}{4}\right)}.$$

(6) $$i\exp_{\kappa }\left(z\right)\equiv \left(\frac{\sqrt{1+\kappa^{2}z^{2}}-\kappa^{2}z}{1-\kappa^{2}}\right)\exp_{\kappa }\left(z\right);$$

(7) $$z=\frac{-\xi^{2}{\left(x-y\right)}^{2}}{4}$$

and $\exp_{\kappa }$ is the deformed exponential function, first introduced by Kaniadakis [1]:

(8) expκ(x)≡(1+κ2x2+κx)1κ.

However, the numerical calculation of Equation (1) can represent a considerable additional amount of computer processing time, especially when inserted in nuclear data processing codes. In order to surpass this issue, Abreu et al. [13] proposed an analytical solution based on obtaining a differential equation and its solution to represent the deformed Doppler broadening function using the Kaniadakis distribution [14]. This analytical solution proved to be up to five times faster than the numerical one [14]. Analytical solutions were also successfully applied in order to obtain faster methods for the Doppler broadening function considering the standard Maxwell-Boltzmann statistics [15] and Tsallis statistics [16].

The validation of the applicability of the Kaniadakis statistic can be performed in other areas through observational data, e.g., cosmic ray flux [12], stellar-residual-radial-velocities [6] and Stellar rotational velocities [4]. However, this kind of approach is not directly applicable to nuclear reactor physics, given the impossibility of observing and measuring the distribution of relative velocities between neutrons and nuclei in a nuclear reactor. Therefore, one of the possible ways to validate the use of κ-statistics is through numerical simulations, similar to in other scientific topics, e.g., relativistic plasmas under the effect of wave-particle interactions [3], non-extensive random matrix theories [17] and Jeans instability of self-gravitating systems [18].

Nevertheless, the accuracy and robustness of the developed solutions contemplating the κ distribution can only be appropriately verified if it is applied in a nuclear data processing code, e.g., FRENDY [19,20], NJOY [21], PREPRO [22] and NECP-Atlas [23]. These systems can process official evaluated nuclear data libraries such as ENDF [24], CENDL [25], JEFF [26] or JENDL [27]. Until the present work, the only results that have been presented for neutron cross-sections considering the Kaniadakis distribution were calculated without doing this [28,29].

Thus, this study’s purpose is to calculate deformed neutron cross-sections of radioactive capture—in the resolved region—for the first time using the Kaniadakis distribution through an analytical solution inside a nuclear data processing code: the FRENDY. 

Additionally, this work aims to apply an alternative numerical methodology to calculate the challenging error functions (with complex arguments) present in the analytical solution of the deformed Doppler broadening function.

## 2. Methodology

The Japan Atomic Energy Agency (JAEA) developed the nuclear data processing code FRENDY (From Evaluated Nuclear Data Library to any application) to treat the most recent nuclear data format, such as the evaluated nuclear library JENDL [27], also developed by JAEA. It was built using the object-oriented language C++ because of its modularity, maintainability, flexibility and portability [30].

Moreover, the FRENDY also intends to work in the future with the recent nuclear data format Generalized Nuclear Data Structure (GNDS), which the current processing codes cannot treat without a considerable amount of format revision [30]. 

To calculate neutron cross-sections in reactor physics, one can use different formalisms such as single-level Breit-Wigner (SLBW), Multi-level Breit-Wigner (MLBW), Adler–Adler and Reich-Moore [30]. To develop the integral formulation for the deformed Doppler broadening function, Equation (1), Guedes et al. [11] used the Single-level one, even though it is not the most recent method. According to the authors, that choice was made because it is easy to implement, it can use published resonance parameters, and it can be Doppler-broadened analytically. It also can be used in reactor physics calculations [11]. Furthermore, the SLBW is the only representation available, for instance, for the ENDF-6 format in the unresolved region [30,31].

### 2.1. Calculating Standard Neutron Cross-Sections with FRENDY

The FRENDY code, by default, uses the Kernel broadening method to more accurately calculate the Doppler-broadened cross-sections in the resolved resonance considering the standard Maxwell-Boltzmann distribution [30]. However, this method demands high computational effort, increasing computational time [30]. Considering this and the fact that the deformed Doppler broadening function using the Kaniadakis statistics adds an extra level of complexity and, consequently, higher computational times, the present work aims to use the single-level Breit-Wigner to calculate the deformed neutron cross-sections in the resolved region. 

One of the advantages of the SLBW method is the possibility of using the ψ−χ method. Through this method, one can represent the standard radiative neutron cross-sections by [30]:

(9) $$\sigma_{\gamma }\left(E,T\right)=\frac{4\pi }{k^{2}}\sum_{j}g_{J}\sum_{r}\Gamma_{\mathrm{nr}}\frac{\Gamma_{\gamma r}}{\Gamma_{r}{}^{2}}\psi \left(\xi ,x\right),$$

$\sigma_{\gamma }$ is the radiative capture cross-section, $\Gamma_{r}$ the total width, $\Gamma_{\gamma r}$ the radiative capture width, $\Gamma_{\mathrm{nr}}$ the neutron widths, k the neutron wave number, $g_{J}$ a spin statistical factor and *E* an incident neutron energy.

To calculate the standard Doppler broadening function, ψ(ξ,x), inside the FRENDY, Tada, Kunieda and Nagaya [30] adopted the four-pole Padé approximation to reduce the calculation time. By using this method, the expression for the standard Doppler broadening function is represented by:

(10) $$\psi \left(s,x\right)≅\frac{\xi \sqrt{\pi }}{2}Re\left[w\left(z\right)\right],$$

where,

(11) $$s=\frac{\xi }{2}\left(x-y\right).$$

w(z) represents a scaled complex complementary error function, commonly known as the Fadeeva or Krump function [32]. It is defined by [33]:

(12) $$w\left(z\right)=e^{-z^{2}}\left[1-erf\left(-iz\right)\right]$$

(13) $$erf\left(z\right)\equiv \frac{2}{\sqrt{\pi }}\int_{0}^{z}e^{-t^{2}}dt,$$

(14) z=u+ih

(15) u=(ξ/2)·x

(16) h=ξ/2

### 2.2. Calculating Deformed Neutron Cross-Sections with FRENDY

As mentioned earlier, the analytical solution of the Doppler broadening function using the Kaniadakis statistics, proposed by Abreu et al. [13] is obtained through a differential equation and its respective resolution [14], given by:

(17) $$\psi_{k}\left(\xi ,x\right)=\Lambda \left(x,\xi \right)\left[D\left(\xi ,x\right)+\Omega_{g}\left(\xi ,x\right)\right],$$

where,

(18) $$\Lambda \left(\xi ,x\right)=\exp\left(\frac{\xi^{2}-\xi^{2}x^{2}}{4}\right)\cdot \frac{\xi \sqrt{\pi }B\left(\kappa \right)}{4};$$

(19) D(ξ,x)≡[Δ(ξ)·cos(Θ)];

(20) $$\Omega_{g}\left(\xi ,x\right)\equiv \Pi \left(x,\xi \right)\cdot \left[i\Omega_{1}\left(\xi ,x\right)+\Omega_{2}\left(\xi ,x\right)\right];$$

(21) Δ(ξ)=2−2erf(ξ2)1−κ2.

(22) $$\Pi \left(\xi ,x\right)=\frac{\sqrt{\xi^{4}-2\xi^{2}\kappa^{2}}}{-\xi^{2}+2\kappa^{2}}\cdot \exp\left(\frac{-\kappa^{2}}{2}\right);$$

(23) $$\Omega_{1}\left(\xi ,x\right)=sin\left(\Theta \right)\cdot \left[erf\left(P_{1}\right)\kappa^{2}-erf\left(P_{1}\right)+erf\left(P_{2}\right)\kappa^{2}-erf\left(P_{2}\right)\right];$$

(24) $$\Omega_{2}\left(\xi ,x\right)=cos\left(\Theta \right)\cdot \left[2erf\left(P_{3}\right)\kappa^{2}-2erf\left(P_{3}\right)-erf\left(P_{1}\right)\kappa^{2}+erf\left(P_{1}\right)+erf\left(P_{2}\right)\kappa^{2}-erf\left(P_{2}\right)\right];$$

(25) $$P_{1}\left(\xi ,x\right)=\frac{-i\xi^{2}x+\sqrt{\xi^{4}-2\xi^{2}\kappa^{2}}}{2\xi };$$

(26) $$P_{2}\left(\xi ,x\right)=\frac{-i\xi^{2}x-\sqrt{\xi^{4}-2\xi^{2}\kappa^{2}}}{2\xi };$$

(27) $$P_{3}\left(\xi ,x\right)=\frac{\sqrt{\xi^{4}-2\xi^{2}\kappa^{2}}}{2\xi };$$

(28) $$\Theta \left(\xi ,x\right)=\frac{x}{2}\sqrt{\xi^{4}-2\xi^{2}\kappa^{2}};$$

To calculate the deformed cross-sections using the Kaniadakis distribution, one needs to substitute the real part of the Faddeeva function for the analytical solution, Equation (17), in the definition of cross-sections in the code, represented by Equation (9), so that:

(29) $$\sigma_{\gamma }\left(E,T\right)=\frac{4\pi }{k^{2}}\sum_{j}g_{J}\sum_{r}\Gamma_{\mathrm{nr}}\frac{\Gamma_{\gamma r}}{\Gamma_{r}{}^{2}}\psi_{\kappa }\left(\xi ,x\right),$$

One of the main challenges in calculating the deformed analytical solution considering the Kaniadakis distribution is calculating the error functions with complex arguments represented in Equations (23) and (24). 

The so-called Gaussian error function, erf(x), is defined as follows [33]:

(30) $$erf\left(x\right)=\frac{2}{\sqrt{\pi }}\int_{0}^{x}e^{-t^{2}}dt.$$

Though these error functions only mean relevance in the tails of the cross-section curves—far from the resonance peak—it is of great significance to implement suitable methodologies to elevate this region’s precision. These regions—due to lower absolute values—usually present the most significant percentual errors. 

The previous works that used the analytical solution implemented the default error functions present in the “special” module of the “scipy” library (Disponible in: https://docs.scipy.org/doc/scipy/reference/special.html accessed on 7 July 2022). However, one cannot find until the date of publishing this manuscript a similar module inside C++; i.e., there is not a unit that directly calculates error functions with complex arguments.

Therefore, the present paper implemented a new methodology to calculate these complex error functions to overcome this problem. The chosen method was the Faddeeva method, developed by Steven Johnson [34]. This methodology has the advantage of using different algorithms to calculate the erf function, Equation (26), according to the value of z. For sufficiently large values of |z|, the package uses a continued-fraction expansion for w(z), analogous to those described by Gautschi [35] and Pope and Wijers [36]. Meanwhile, for smaller values of |z| or for z close to the real axis [34], Johnson used the algorithm 916, developed by Zaghloul and Ali [34]. According to Johnson, “algorithm 916 is competitive and faster for smaller values of |z| and also has better relative accuracy in Re[z] for some regions near the real-z-axis” [34].

In fact, by using the Faddeeva method to calculate the complex error functions inside the deformed analytical Doppler broadening function, $\psi_{\kappa }\left(\xi ,x\right)$, presented more accurate results in the tail region. In the next section, these results will be shown. 

After conducting this modification, we used the FRENDY to calculate the deformed radiative capture neutron cross-section. Initially, we calculated the deformed cross-sections using the same adopted range of energy in the JENDL-4.0 library [27]. After that, two different resonance peaks were selected in order to compare with the results considering the Maxwell–Boltzmann distribution. Four nuclides in JENDL-4.0 are considered. The major calculation conditions are summarized as follows:

- Method: single-level Breit-Wigner;
- Nuclides: Pu238, Tc99, Gd155 and Gd157;
- Temperatures (K): 1500, 2000 and 2500;
- Maximum number of points (h_max): 10,000;
- Range of energy: 10^−2^ to 10^7^ eV;
- Deformation in relation to the MB distribution: *κ* = 0.1.

## 3. Results and Discussion

Calculating the numerical deformed Doppler broadening function using the Kaniadakis entropy can be very computationally costly. In fact, in a recently published paper [14], the analytical solution provided by Equation (9) was approximately 4.6 times faster than the numerical one. 

**Figure 1:**
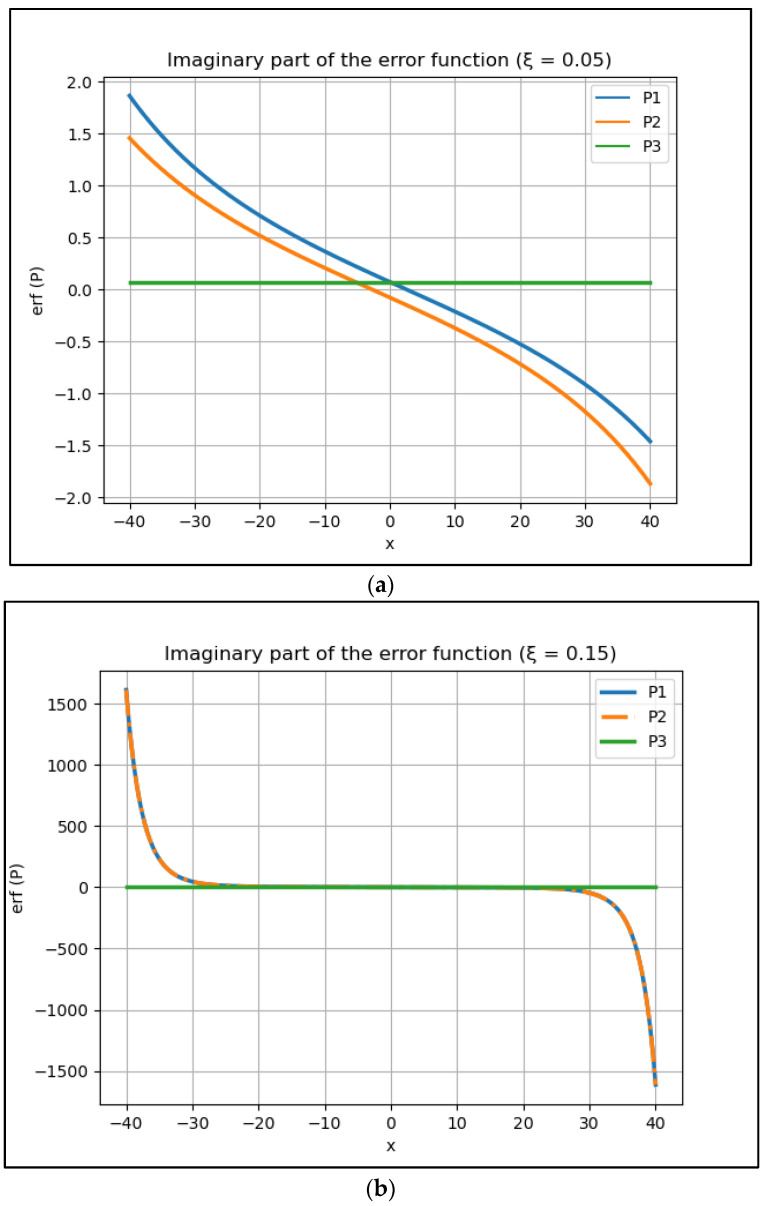
The imaginary part of the error function for P1, P2 and P3 considering two different values of ξ: (**a**) = 0.05 and (**b**) = 0.15. The real part of these error functions is close to zero ($≅10^{-18}$ ) and, consequently, presents little relevance.

**Figure 2:**
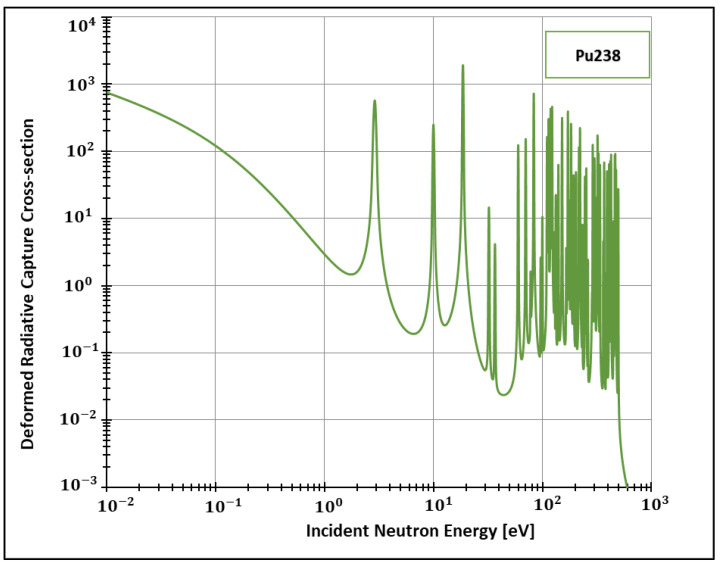
Deformed Radiative capture cross-section for Plutonium 238 considering 1500 K and k=0.1.

However, the analytical solution represented by Equation (9) presents higher values of percentual errors to the curve’s tail, far from the resonance peak. These higher values are linked to the small values in these regions and the fact that the error functions, including those for P1 and P2, Equations (25) and (26), tend to present a more significant influence in these specific regions, as one can see in Figure 1 and Figure 2.

Consequently, the application of a new, more robust method for the calculation of these functions could present an improvement to the deformed Doppler broadening functions. In fact, the Faddeeva package results showed better numbers, as seen in Table 1, Table 2 and Table 3, representing the percentual error of the analytical solution in relation to the numerical one.

By analyzing Table 3, it is possible to note the lower values of percentual error when one uses the Faddeeva method to calculate the existing error functions in the deformed analytical solution of the Doppler broadening function using the Kaniadakis entropy. The maximum percentual reduction was 2.5%.

### Deformed Cross-Sections with FRENDY

After implementing the deformed analytical solution for the Doppler broadening function inside FRENDY’s test module, we were able to generate data for the deformed radiative cross-sections for different elements. Considering the adopted method for calculating these quantities (SLBW), FRENDY’s default package offers the calculation of cross-sections for two important elements: Plutonium 238 and Technetium 99. The former element (Pu238) is of crucial importance, for instance, to space exploration [37] and Mars colonization [38,39]. In addition, 80% of the scans performed in nuclear medicine departments are made from the latter element [40]. Both can be produced in research nuclear reactors, such as the High Flux Isotope Reactor in the United States [41] and the Moly project of the recent Research Reactor Jules Horowitz (JHR), still under construction in France [40].

**Figure 3:**
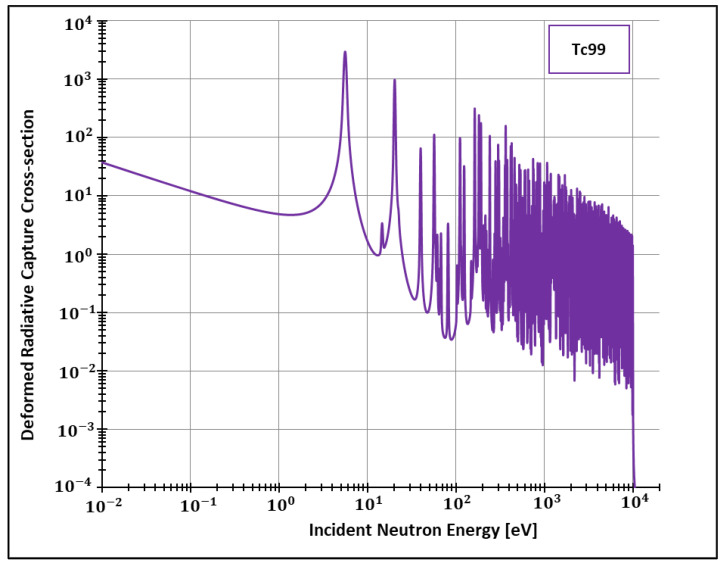
Deformed Radiative capture cross-section for Technetium 99 considering 1500 K and k=0.1.

**Figure 4:**
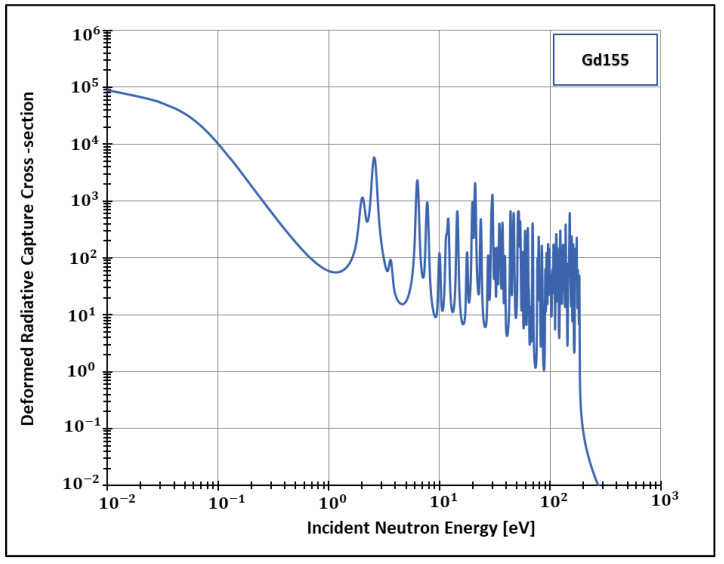
Deformed Radiative capture cross-section for Gadolinium 155 considering 1500 K and k=0.1.

**Figure 5:**
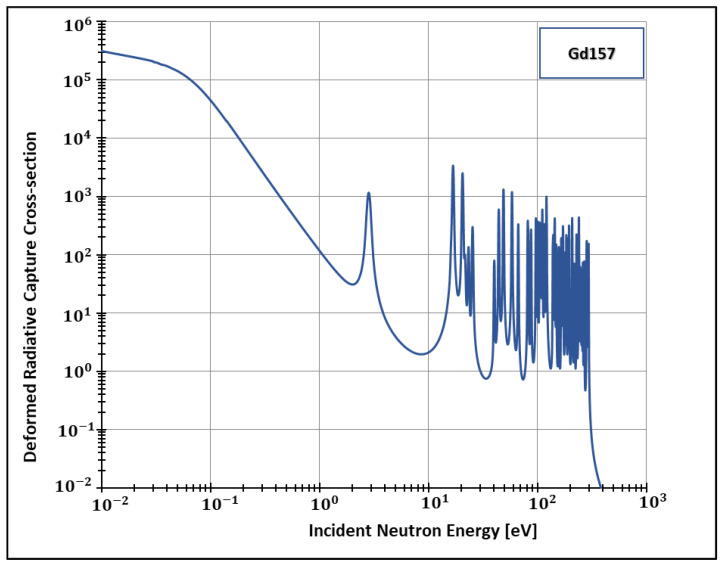
Deformed Radiative capture cross-section for Gadolinium 157 considering 1500 K and k=0.1.

Additionally, the present work generated data for the isotopes 155 and 157 (Figure 2, Figure 3, Figure 4 and Figure 5) of gadolinium, which is widely used for medical applications [42], radiation shielding [43], and also space exploration [44].

**Figure 6:**
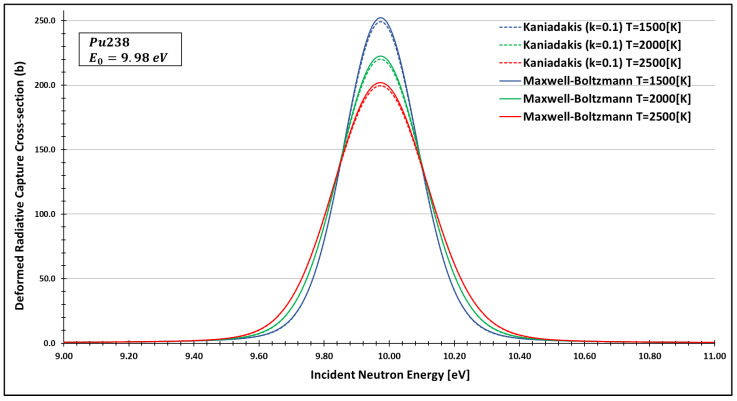
Deformed Radiative capture cross-section for Plutonium 238 considering k=0.1 and the 9.98 eV peak.

**Figure 7:**
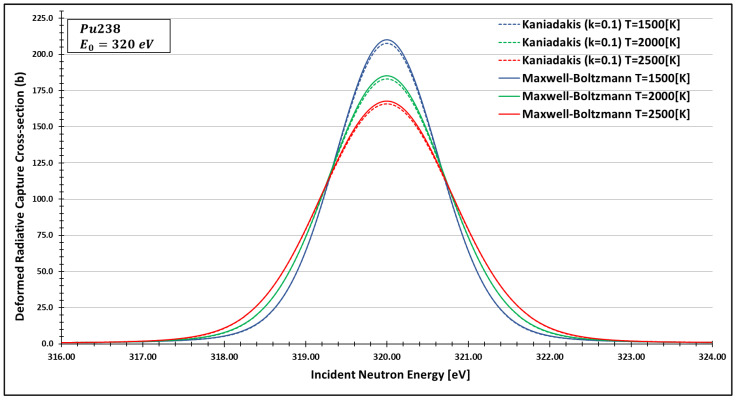
Deformed Radiative capture cross-section for Plutonium 238 considering k=0.1 and the 320 eV peak.

**Figure 8:**
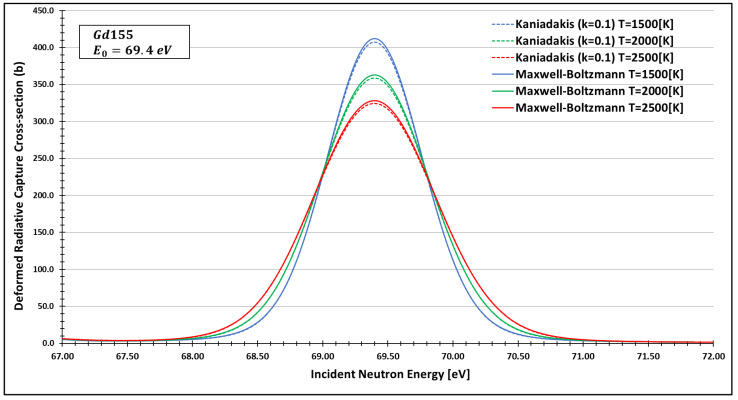
Deformed Radiative capture cross-section for Gadolinium 155 considering k=0.1 and the 69.4 eV peak.

**Figure 9:**
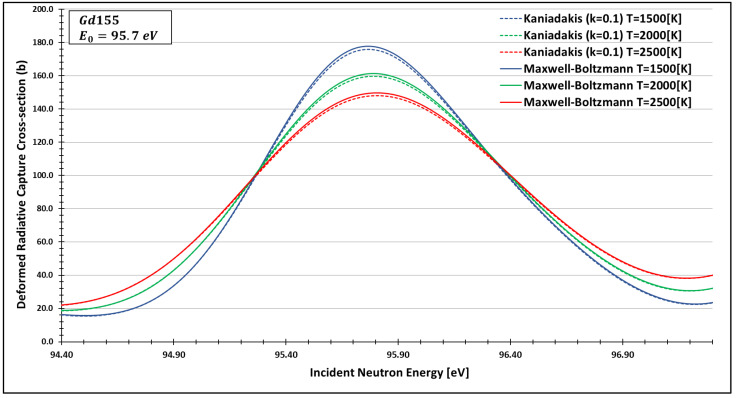
Deformed Radiative capture cross-section for Gadolinium 155 considering k=0.1 and the 95.7 eV peak.

**Figure 10:**
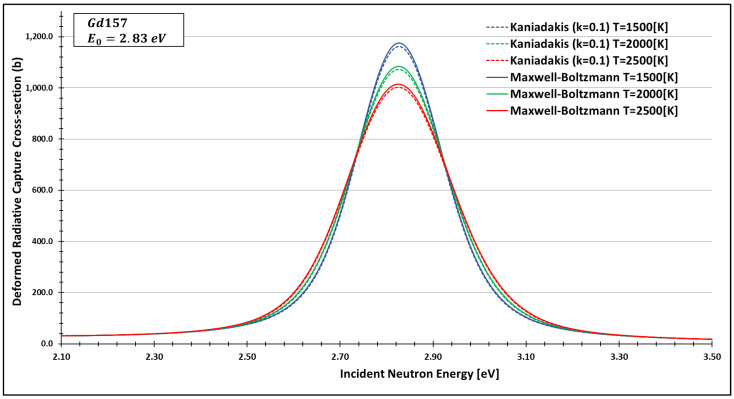
Deformed Radiative capture cross-section for Gadolinium 157 considering k=0.1 and the 2.83 eV peak.

**Figure 11:**
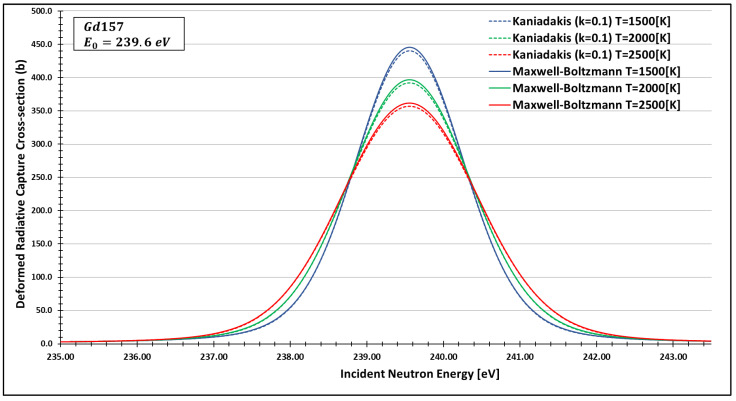
Deformed Radiative capture cross-section for Gadolinium 157 considering k=0.1 and the 239.6 eV peak.

**Figure 12:**
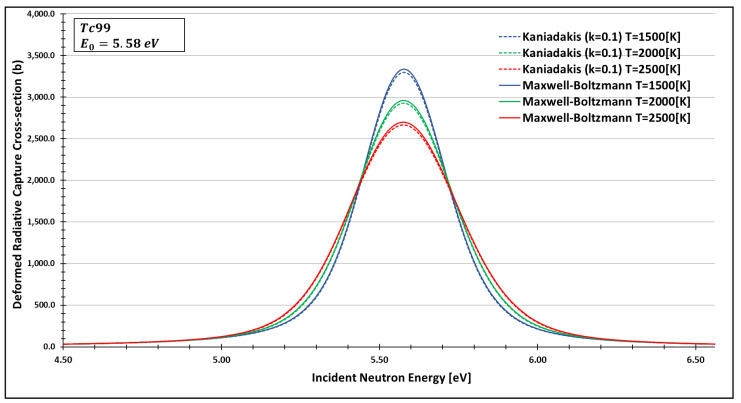
Deformed Radiative capture cross-section for Technetium 99 considering k=0.1 and the 5.58 eV peak.

**Figure 13:**
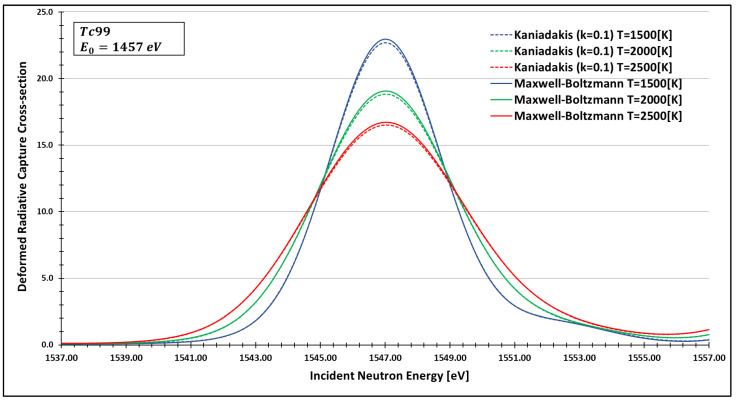
Deformed Radiative capture cross-section for Technetium 99 considering k=0.1 and the 1457 eV peak.

In order to see more closely and compare the standard Maxwell-Boltzmann behavior with the Kaniadakis, we selected two different resonance peaks—apart from each other—at three different temperatures (Figure 6, Figure 7, Figure 8, Figure 9, Figure 10, Figure 11, Figure 12 and Figure 13) to confirm the expected curve attenuation illustrated in previous works [13,14,29,45]:

As one can see, the deformed curves presented the expected behavior since there is an attenuation of the resonance curves, especially on the peaks, compared to the standard neutron radiative cross-section using the Maxwell–Boltzmann entropy. In fact, the relative error between the Maxwell–Boltzmann and Kaniadakis peaks is around 1%, which is the same order of magnitude (~1%) obtained in previous works for the calculations of deformed Doppler broadening functions using the Kaniadakis entropy.

## 4. Concluding Remarks

After 20 years of development of the Kaniadakis entropy and seven years of its application in nuclear reactor physics, this work presents for the first-time results for deformed neutron cross-sections considering the κ statistics using an official nuclear data processing code, FRENDY, and, consequently, official nuclear data (JENDL 4.0). This work was carried out by implementing the analytical solution for the deformed Doppler function using the Kaniadakis statistics, $\psi_{\kappa }$, inside the single-level Breit–Wigner module in the FRENDY. We used MIT’s Faddeeva method to calculate the error functions inside the analytical solution. This implementation showed a percentual error reduction when compared to the numerical solution of $\psi_{\kappa }$. 

With the implementation of $\psi_{\kappa }$ inside FRENDY, it was possible to calculate deformed radiative capture cross-sections for four relevant nuclides: Pu238, Gd 155, Gd 157 and Tc99. Next, we selected two different resonance peaks of each nuclide to compare the data with the standard Maxwell-Boltzmann curves generated by the FRENDY code. The results agreed with previous calculations conducted out of a nuclear data processing code and without official nuclear data libraries. 

Different from other areas, the evaluation of the viability of Kaniadakis entropy in the area of the nuclear reactor physics cannot be conducted observationally. Therefore, it is of great relevance to implement this methodology in nuclear data processing codes where it is possible to deal with accurate data. Thus, the present work can be interpreted as an essential step in validating the applicability of the Kaniadakis entropy in the nuclear fission area. 

## Figures and Tables

**Table 1:** The percentual errors of the analytical solution in relation to the numerical one using the Numpy package (Python) to calculate the included error functions.

ξ	x = 0	x = 0.5	x = 1	x = 2	x = 4	x = 6	x = 8	x = 10	x = 20	x = 40
0.05	0.02	0.02	0.00	0.02	0.02	0.02	0.02	0.02	0.06	0.67
0.10	0.04	0.04	0.04	0.04	0.05	0.05	0.06	0.09	0.73	12.55
0.15	0.06	0.06	0.06	0.06	0.07	0.11	0.17	0.32	3.77	10.39
0.20	0.08	0.08	0.08	0.09	0.12	0.21	0.44	0.85	9.60	4.48
0.25	0.10	0.09	0.10	0.11	0.19	0.41	0.90	1.89	10.91	3.08
0.30	0.11	0.11	0.12	0.15	0.29	0.71	1.67	3.50	6.89	3.17
0.35	0.13	0.13	0.15	0.19	0.44	1.16	2.78	5.46	5.00	4.84
0.40	0.15	0.15	0.17	0.23	0.63	1.75	4.09	7.04	4.09	3.23
0.45	0.17	0.18	0.19	0.29	0.87	2.51	5.42	7.64	3.82	3.23
0.50	0.19	0.20	0.22	0.36	1.18	3.37	6.41	7.32	3.49	3.28

**Table 2:** The percentual errors of the analytical solution in relation to the numerical one using the Faddeeva package (C++) to calculate the included error functions.

ξ	x = 0	x = 0.5	x = 1	x = 2	x = 4	x = 6	x = 8	x = 10	x = 20	x = 40
0.05	0.02	0.02	0.02	0.02	0.02	0.02	0.02	0.02	0.06	0.67
0.10	0.04	0.04	0.04	0.04	0.04	0.05	0.06	0.08	0.68	10.61
0.15	0.06	0.06	0.06	0.06	0.07	0.09	0.15	0.26	3.40	8.00
0.20	0.08	0.08	0.08	0.08	0.10	0.17	0.34	0.71	8.17	3.08
0.25	0.10	0.10	0.10	0.10	0.15	0.31	0.73	1.59	8.89	2.55
0.30	0.11	0.11	0.12	0.13	0.22	0.55	1.37	2.96	5.73	2.41
0.35	0.13	0.13	0.14	0.16	0.32	0.89	2.28	4.55	3.72	2.34
0.40	0.15	0.15	0.16	0.19	0.45	1.37	3.36	5.82	2.97	2.30
0.45	0.17	0.17	0.18	0.23	0.63	1.97	4.42	6.28	2.68	2.28
0.50	0.19	0.19	0.20	0.27	0.85	2.63	5.18	5.92	2.54	2.26

**Table 3:** The percentual difference between Table 1 and Table 2.

ξ	x = 0	x = 0.5	x = 1	x = 2	x = 4	x = 6	x = 8	x = 10	x = 20	x = 40
0.05	0.00	0.00	0.02	0.00	0.00	0.00	0.00	0.00	0.00	0.01
0.10	0.00	0.00	0.00	0.00	0.01	0.01	0.00	0.01	0.05	1.94
0.15	0.00	0.00	0.00	0.00	0.01	0.02	0.03	0.06	0.38	2.39
0.20	0.00	0.00	0.00	0.01	0.02	0.04	0.09	0.14	1.44	1.40
0.25	0.00	0.00	0.00	0.01	0.04	0.09	0.17	0.30	2.01	0.53
0.30	0.00	0.00	0.00	0.02	0.07	0.16	0.30	0.54	1.16	0.77
0.35	0.00	0.00	0.01	0.03	0.12	0.26	0.50	0.90	1.28	2.50
0.40	0.00	0.00	0.01	0.04	0.18	0.38	0.73	1.21	1.12	0.92
0.45	0.00	0.01	0.02	0.06	0.24	0.55	1.01	1.37	1.14	0.95
0.50	0.00	0.01	0.02	0.09	0.33	0.73	1.23	1.40	0.95	1.02
